# Current status of 5-aminosalicylic acid in Crohn’s disease treatment: a nationwide survey and analysis of perspectives and practice patterns among inflammatory bowel disease physicians

**DOI:** 10.3389/fgstr.2026.1783772

**Published:** 2026-04-27

**Authors:** Yuanli Li, Changxin Chen, Chengdang Wang

**Affiliations:** 1 Department of Gastroenterology Endoscopy Center, Quanzhou First Hospital, Quanzhou, Fujian, China; 2 Department of Gastroenterology, The First Affiliated Hospital of Fujian Medical University, Fuzhou, Fujian, China

**Keywords:** 5-aminosalicylic acid, Crohn’s disease, perspective, physician, questionnaire survey

## Abstract

**Objective:**

To investigate the perspectives and clinical practice patterns of inflammatory bowel disease (IBD) physicians regarding the use of 5-aminosalicylic acid (5-ASA) in Crohn’s disease (CD).

**Methods:**

A 21-item questionnaire was distributed to IBD physicians across various regions in mainland China via WeChat. The survey evaluated prescription frequencies, indications, dosages, and physician rationales. The questionnaire was pilot-tested among 30 clinicians of varying seniority levels to ensure clarity and content validity. Responses were anonymized, and duplicate submissions were prevented through WeChat account verification. Statistical analysis employed the chi-square test.

**Results:**

Among 615 respondents, 90.4% reported prescribing 5-ASA for CD patients. It was primarily used for mild disease (91.7%), colonic involvement (77.9%), and non-stricturing, non-penetrating phenotypes (91.4%). For induction therapy, 58.1% of physicians utilized 5-ASA, with 89.2% employing it in combination regimens (predominantly with corticosteroids, 90.3%). Furthermore, 83.1% prescribed 5-ASA for maintenance therapy. The primary rationales cited were its favorable safety profile (75.5%) and perceived efficacy in mild cases (68.2%).

**Conclusions:**

Despite unequivocal evidence and international guidelines recommending against the routine use of 5-ASA in CD, it remains pervasively prescribed by Chinese clinicians. This highlights a profound discrepancy between evidence-based guidance and real-world practice. Such therapeutic inertia risks delaying the initiation of highly effective therapies, underscoring an urgent need for targeted continuing medical education to optimize CD management.

## 1 Introduction

Crohn’s disease (CD) is a chronic, idiopathic inflammatory bowel disorder characterized by relapsing and remitting inflammation, typically progressive in nature, and currently incurable. Following medically-induced remission or surgery, long-term maintenance therapy with various agents is required to sustain remission. Current therapeutic options for CD include corticosteroids, immunosuppressants, and biological agents (1). It is crucial to strictly differentiate the therapeutic role of 5-ASA in ulcerative colitis (UC) from its application in CD. In UC, 5-ASA maintains a robust, undisputed evidence base for both induction and maintenance therapies (2, 3). Conversely, in CD, the same class of medication has repeatedly failed to demonstrate meaningful, reproducible clinical benefits in altering the natural disease course (4). Extensive randomized controlled trials (RCTs) and subsequent meta-analyses have shown that 5-ASA is no more effective than placebo for inducing or maintaining remission in CD (5–8). Consequently, major international guidelines, including those from the European Crohn’s and Colitis Organisation (ECCO), the American College of Gastroenterology (ACG), and the British Society of Gastroenterology (BSG), do not recommend 5-ASA for CD (9–12). However, real-world studies in Western countries indicate that a significant proportion of clinicians continue to prescribe 5-ASA for CD (13, 14). Currently, data on Chinese clinicians’ perspectives regarding 5-ASA use in CD are lacking. Therefore, this study aimed to investigate the views of IBD-focused clinicians in mainland China on the use of 5-ASA for CD and to quantify the evidence-practice gap in this setting.

## 2 Objects and methods

### 2.1 Questionnaire design and validation

The questionnaire was designed by the research team based on a literature review and input from three senior IBD specialists. To ensure content validity, the initial draft of the questionnaire underwent expert review by a panel of senior IBD specialists. The questionnaire was pilot-tested among 30 clinicians of varying seniority levels (residents, attending physicians, associate chief physicians, and chief physicians) to assess clarity, comprehensibility, and relevance. Feedback from the pilot test was used to refine question wording and response options. The final questionnaire consisted of 21 items, including single-choice and multiple-choice questions, covering: physician demographics; frequency of 5-ASA prescription; formulations and dosages prescribed; use of combination therapy; patient adherence; perceived side effects of 5-ASA; and reasons supporting or opposing its use (see **Supplementary Material**). It is important to note that the questionnaire did not provide a standardized definition or scoring index (e.g., CDAI) for “mild CD.” Instead, respondents were asked to base their answers on their own subjective clinical judgment of “mild disease, “ which reflects real-world diagnostic heterogeneity.

### 2.2 Survey method and data collection

Questionnaires were distributed via WeChat to IBD association groups across provinces, municipalities, and autonomous regions in mainland China between March and June 2023. The survey purpose, response method and precautions were clearly explained. Participation was anonymous, with confidentiality assured. Respondents were instructed that there were no “correct answers” and to answer objectively based on their actual clinical practice. Physicians completed questionnaires independently without discussion; any ambiguities were clarified by the research team. To prevent duplicate submissions, we enabled the “one response per WeChat account” setting. To minimize clustering bias, we limited the number of responses from any single institution to a maximum of five. This survey study was reviewed by the Ethics Committee of Quanzhou First Hospital and was granted an exemption from requiring formal ethical approval because it involved anonymous questionnaires for healthcare professionals with no personal identifiable information collected.

### 2.3 Statistical analysis

Questionnaires that were fully completed and contained no contradictory responses were deemed valid and included in the analysis. Data were analyzed using SPSS version 25.0. Descriptive statistics were employed. Categorical data were presented as percentages. Comparisons between rates were performed using the chi-square test or Fisher’s exact test, as appropriate. Multiple comparisons utilized Bonferroni correction. Statistical significance was defined as P < 0.05.

## 3 Results

### 3.1 Physician demographics

A total of 615 valid questionnaires were collected. The response rate was approximately 68% based on the estimated number of physicians in the WeChat groups (approximately 900 members). Demographic characteristics are summarized in **Table 1**.

**Table 1:** Physician demographics.

Characteristic	N=615(%)
Hospital level	
Univer-affiliated Tertiary A	259(42.1)
Non- university Tertiary A	166(27.0)
Tertiary B	45(7.3)
Secondary A	122(19.9)
Other	23(3.7)
Specialty	
Gastroenterology	596(96.9)
Colorectal surgery	4(0.7)
Pediatrics	4(0.7)
Other	11(1.8)
Professional title	
Chief Physician	162(26.3)
Associate Chief Physician	229(37.2)
Attending Physician	167(27.2)
Resident Physician	57(9.3)
Years Focus on IBD	
≤5 years	202(32.8)
>5–10 years	168(27.3)
>10–15 years	113(18.4)
>15–20 years	68(11.1)
More than 20 years	64(10.4)
Area	
North China	45(7.3)
Northeast China	3(0.5)
East China	229(37.2)
Central China	93(15.1)
South China	82(13.4)
Southwest China	93(15.1)
Northwest China	70(11.4)

### 3.2 Utilizations of 5-ASA in CD

#### 3.2.1 Perspectives on the efficacy of 5-ASA for CD

Among the 615 respondents, 556 (90.4%) reported prescribing 5-ASA.

Regarding perceived efficacy, 338 physicians (55%) believed 5-ASA was effective for CD, 218 (35.4%) believed it might be effective, and only 59 (9.6%) believed it was ineffective (**Supplementary Figure 1**).

#### 3.2.2 Types of CD for which 5-ASA is prescribed

Among the 556 physicians prescribing 5-ASA: 323 (58.1%) used it for induction therapy, 442 (79.5%) used it for maintenance therapy following medically-induced remission, 212 (38.1%) used it for postoperative maintenance therapy, 54 (9.7%) used it for other indications (**Supplementary Figure 2**).

Regarding disease severity: 510 physicians (91.7%) prescribed it for mild CD, 317 (57%) for moderate CD, 87 (15.6%) for severe CD (**Supplementary Figure 2**).

Regarding disease location: 433 physicians (77.9%) prescribed it for colonic disease (L2), 361 (64.9%) for ileal disease (L1), 318 (57.2%) for ileocolonic disease (L3), 57 (10.3%) for upper gastrointestinal involvement (L4) (**Supplementary Figure 2**).

Regarding disease behavior: 508 physicians (91.4%) prescribed it for non-stricturing non-penetrating disease (B1), 158 (28.4%) for stricturing disease (B2), 84 (15.1%) for penetrating disease (B3) (**Supplementary Figure 2**).

#### 3.2.3 5-ASA formulations prescribed

Among the 556 physicians prescribing 5-ASA (This was a multiple-choice question; therefore, the sum of percentages exceeds 100%.): 369 (66.4%) prescribed mesalamine enteric-coated tablets, 296 (53.2%) prescribed mesalamine extended-release granules, 274 (49.3%) prescribed mesalamine extended-release tablets, 191 (34.4%) prescribed sulfasalazine, 33 (5.9%) prescribed olsalazine, 26 (4.7%) prescribed balsalazide (**Supplementary Figure 3**).

#### 3.2.4 Dosage and combination therapy for induction of remission

**Figure 1:**
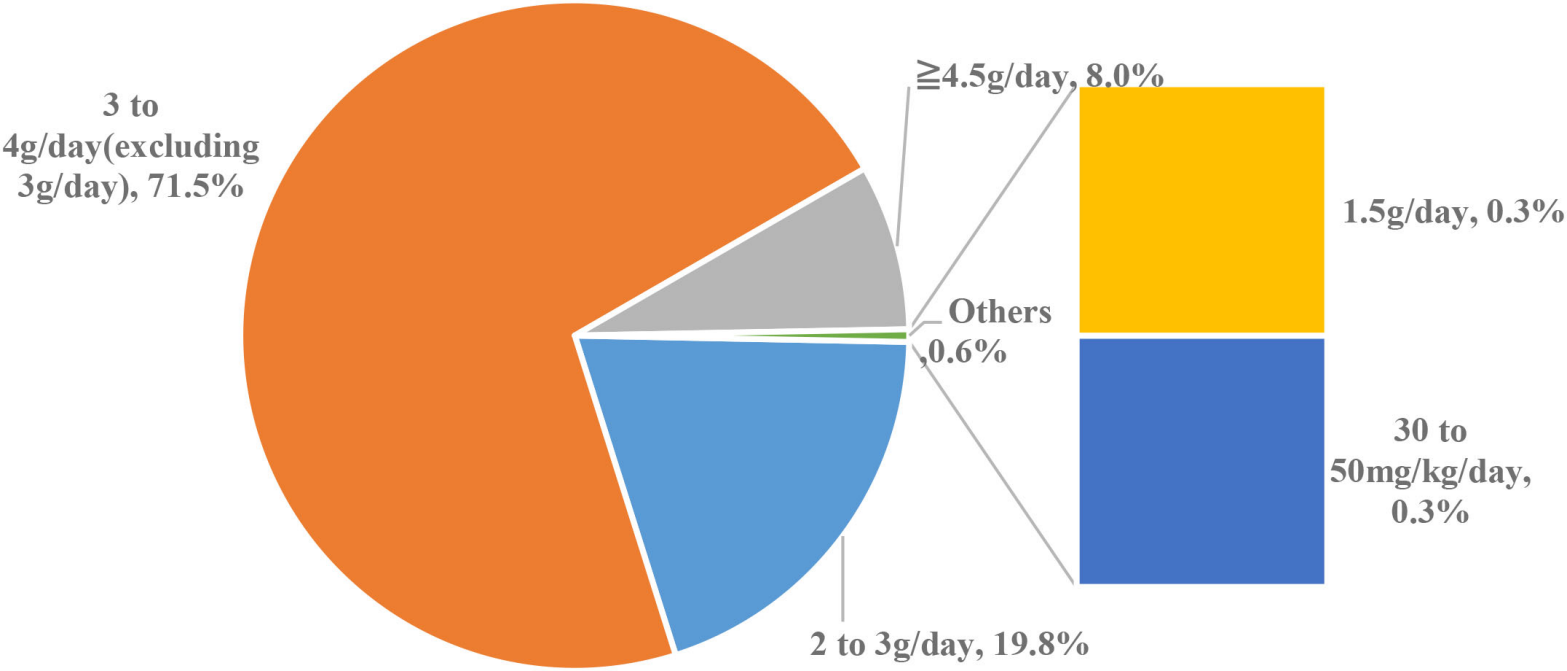
Dosage of 5-ASA prescribed for induction of remission.

Among the 323 physicians using 5-ASA for induction therapy: Dosage: 26 physicians (8%) prescribed ≥4.5 g/day; 231 (71.5%) prescribed 3–4 g/day(excluding 3 g/day); 64 (19.8%) prescribed 2–3 g/day; 1 (0.3%) prescribed 1.5 g/day; 1 (0.3%) prescribed based on weight (30–50 mg/kg/day) (**Figure 1**).

Combination Therapy: 151 physicians (46.7%) decided on combination therapy case-by-case; 137 (42.4%) always used combination therapy; 35 (10.8%) did not use combination therapy.

**Figure 2:**
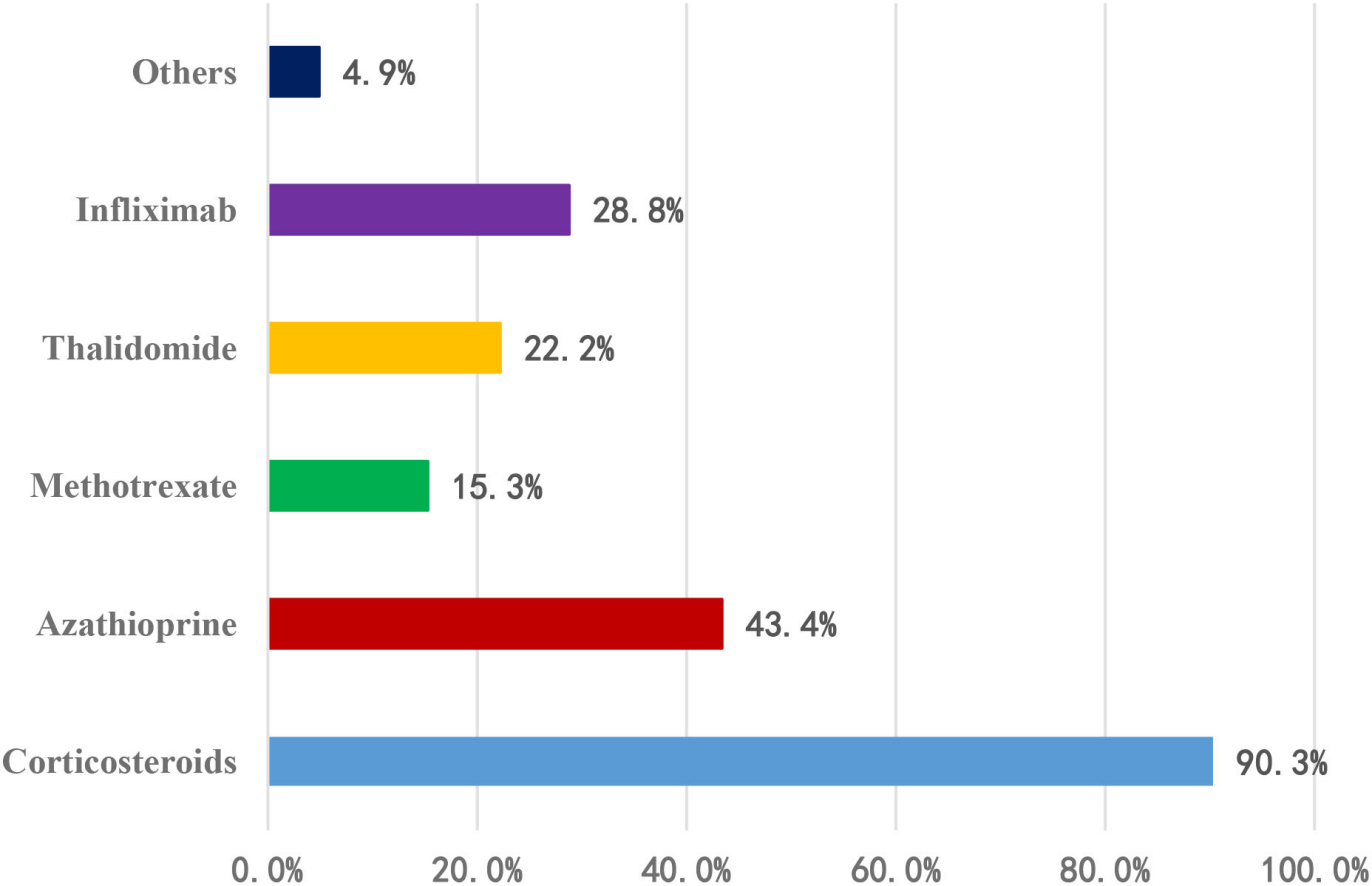
Combination therapy regimens for induction of remission (This was a multiple-choice question; therefore, the sum of percentages exceeds 100%).

Among the 288 physicians using combination therapy (This was a multiple-choice question; therefore, the sum of percentages exceeds 100%.): 260 (90.3%) combined with corticosteroids; 125 (43.4%) with azathioprine; 83 (28.8%) with infliximab; 64 (22.2%) with thalidomide; 44 (15.3%) with methotrexate; 14 (4.9%) with other agents (**Figure 2**).

#### 3.2.5 Dosage and combination therapy for maintenance therapy

A total of 462 physicians considered using 5-ASA for maintenance therapy.

**Figure 3:**
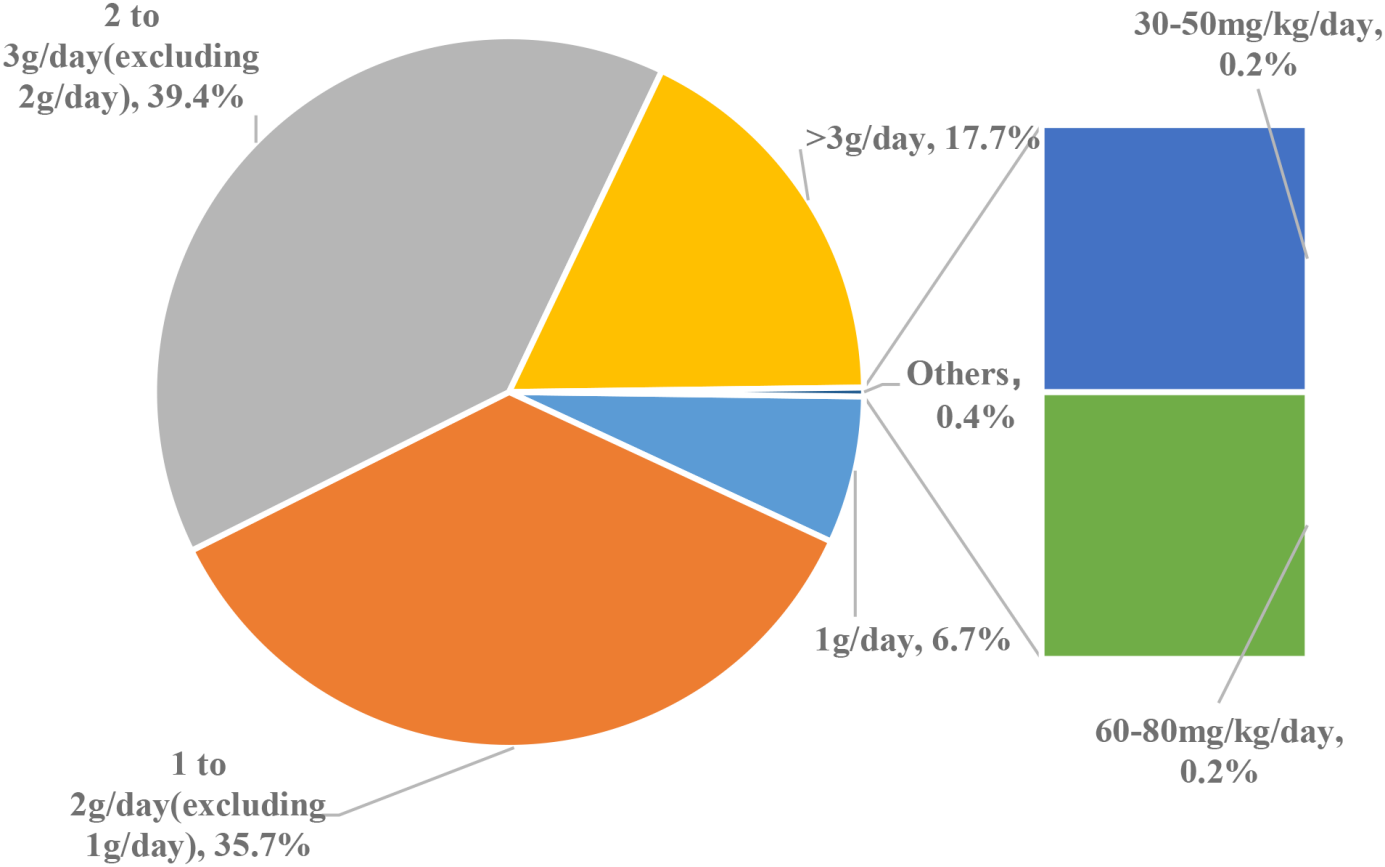
Dosage of 5-ASA prescribed for maintenance therapy.

Dosage: 82 physicians (17.7%) prescribed >3 g/day; 182 (39.4%) prescribed 2–3 g/day (excluding 2 g/day); 165 (35.7%) prescribed 1–2 g/day(excluding 1 g/day); 31 (6.7%) prescribed 1 g/day; 1 (0.2%) prescribed based on weight (30–50 mg/kg/day); 1 (0.2%) prescribed based on weight (60–80 mg/kg/day) (**Figure 3**).

Combination Therapy: 217 physicians (47.0%) decided on combination therapy case-by-case; 94 (20.3%) always used combination therapy; 151 (32.7%) used 5-ASA monotherapy.

**Figure 4:**
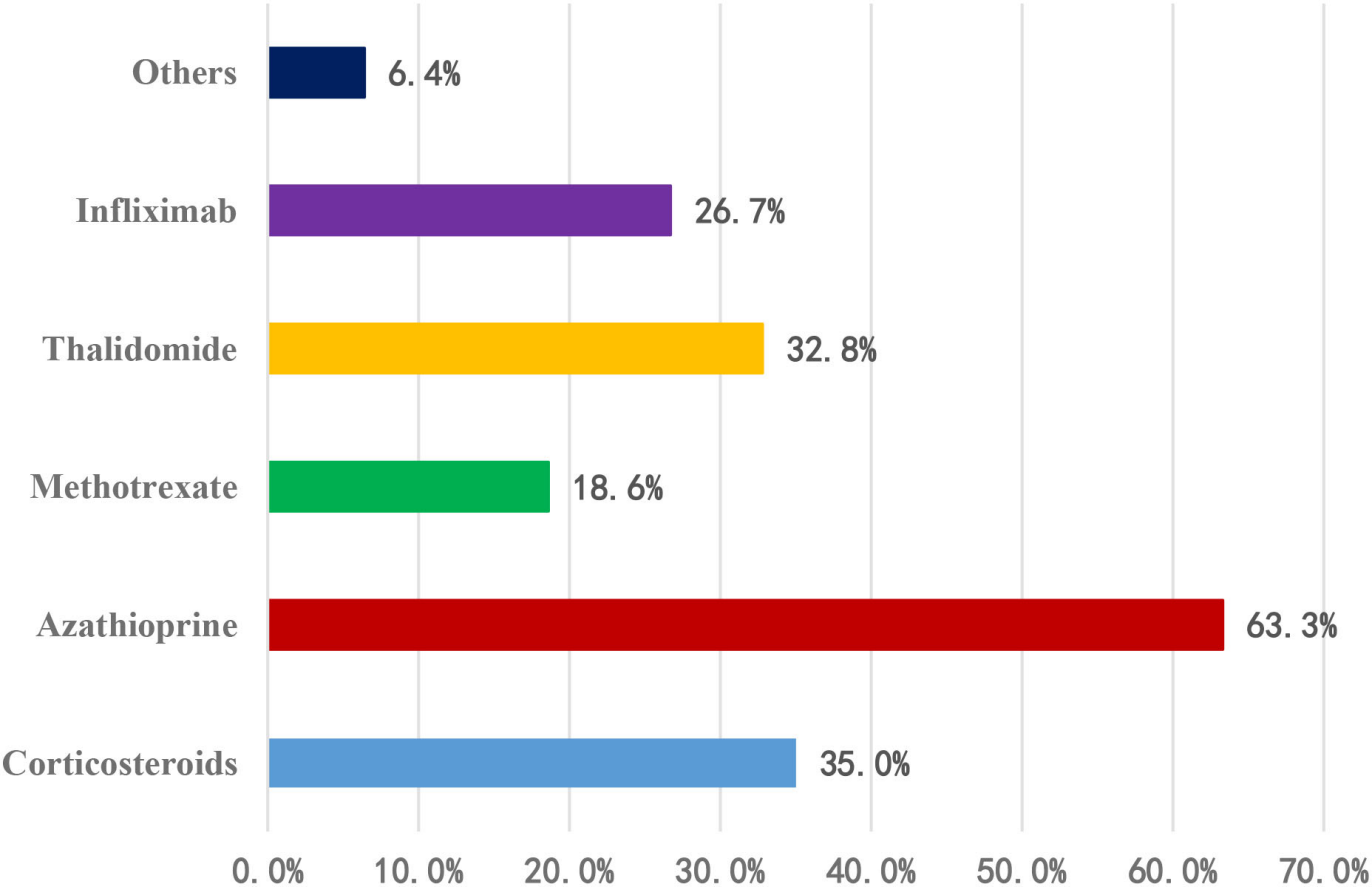
Combination therapy regimens for maintenance therapy (This was a multiple-choice question; therefore, the sum of percentages exceeds 100%).

Among the 311 physicians using combination therapy (This was a multiple-choice question; therefore, the sum of percentages exceeds 100%.): 197 (63.3%) combined with azathioprine; 109 (35.0%) with corticosteroids; 102 (32.8%) with thalidomide; 83 (26.7%) with infliximab; 58 (18.6%) with methotrexate; 20 (6.4%) with other agents (**Figure 4**).

#### 3.2.6 Adverse events of 5-ASA reported by physicians

Regarding perceived adverse events: 346 physicians (62.2%) identified gastrointestinal reactions (e.g., abdominal pain, diarrhea) as the most common. 292 (52.5%) reported abnormal liver function. Only 12 physicians reported no significant adverse events encountered to date. Other reported events included pancreatitis (n=3), arthralgia (n=2), and alopecia (n=1) (**Table 2**).

**Table 2:** Adverse events of 5-ASA reported by physicians.

Projects	N(%)
Gastrointestinal (e.g., abdominal pain and diarrhea)	346(62.2)
Abnormal liver function	292(52.5)
Rash	215(38.7)
Neurological (e.g., dizziness, headache)	175(31.5)
Hematological issues	161(29.0)
Allergy	129(23.2)
Renal impairment	106(19.1)
Others	55(9.9)

This was a multiple-choice question; therefore, the sum of percentages exceeds 100%).

#### 3.2.7 Physicians’ assessment of patient adherence to 5-ASA at 6 months

**Figure 5:**
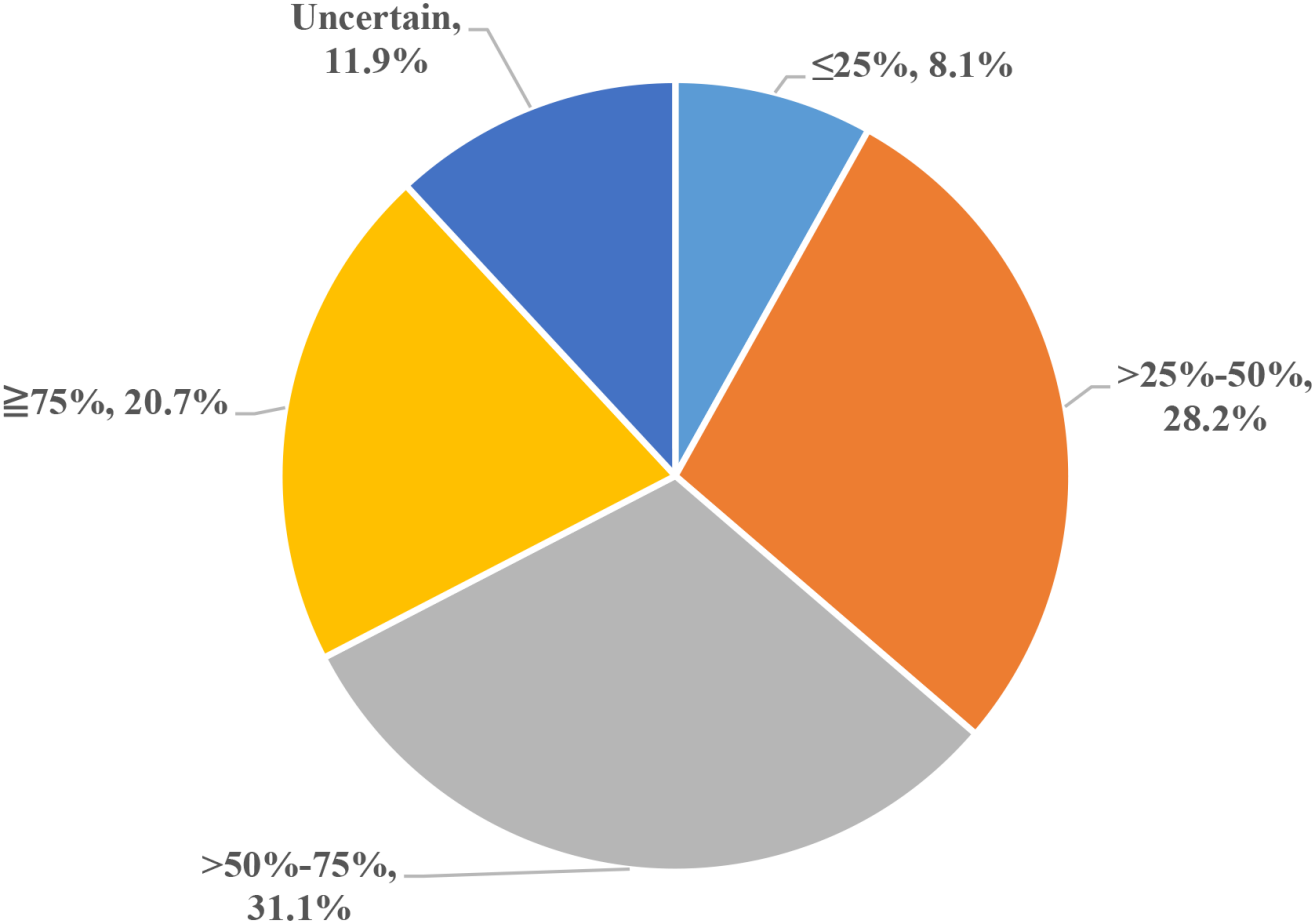
Physicians’ assessment of patient adherence to 5-ASA at 6 months.

Regarding patient adherence to 5-ASA for ≥6 months: 115 physicians (20.7%) estimated adherence ≥75%. 173 (31.1%) estimated adherence >50-75%. 157 (28.2%) estimated adherence >25-50%. 45 (8.1%) estimated adherence ≤25%. 66 (11.9%) were uncertain about adherence levels (**Figure 5**).

#### 3.2.8 Reasons physicians support 5-ASA use in CD

The 556 physicians supporting 5-ASA use cited the following reasons: Good safety profile and low toxicity (420 physicians, 75.5%). Perceived efficacy in mild CD and patient subgroups not subjected to RCTs (379 physicians, 68.2%). Relatively low cost, particularly compared to biologics (351 physicians, 63.1%). Potential prevention of IBD-associated colorectal cancer (189 physicians, 34.0%). Personal clinical experience suggesting efficacy (188 physicians, 33.8%).

#### 3.2.9 Reasons physicians do not support 5-ASA use in CD

The 59 physicians not supporting 5-ASA use cited: High-quality evidence showing minimal benefit over placebo for induction or maintenance (51 physicians, 86.4%). Belief that other therapies offer higher remission rates and improved quality of life (32 physicians, 54.2%). Concern over rare but serious adverse events limiting use (3 physicians, 5.1%). Perception of relatively high cost (1 physician, 1.7%).

### 3.3 Perspectives on 5-ASA for CD treatment across different physician groups

#### 3.3.1 Perspectives on efficacy across groups

Hospital Level: Efficacy beliefs varied significantly by hospital level (X²=36.767, P = 0.001). *Post-hoc* comparisons (Bonferroni) indicated a significant difference between university-affiliated tertiary A hospitals and secondary A (X²=34.192, P<0.001), suggesting lower perceived efficacy/usage in higher-level hospitals. See **Table 3** for details.

Professional Title: Efficacy beliefs varied significantly by title (X²=13.4, P = 0.037). See **Table 3**.

Years Focused on IBD: No significant difference in efficacy perspectives based on years of IBD experience (X²=13.44, P>0.05). See **Table 3**.

**Table 3:** Perspectives on efficacy of 5-ASA for CD treatment across physician groups.

Projects	Effectiveness(%)	Possibly(%)	Ineffective(%)	X²	P
Hospital level					
Univer-affiliated Tertiary A	113(43.6)	111(42.9)	35(13.5)	36.767	0.001
Non- university Tertiary A	93(56.1)	60(36.1)	13(7.8)		
Tertiary B	28(62.2)	13(28.9)	4(8.9)		
Secondary A	92(75.4)	25(20.5)	5(4.1)		
Other	12(52.2)	9(39.1)	2(8.7)		
Professional title					
Chief physician	83(51.2)	69(42.6)	10(6.2)	13.4	0.037
Associate chief physician	125(54.6)	77(33.6)	27(11.8)		
Attending physician	89(53.3)	59(35.3)	19(11.4)		
Resident physician	41(71.9)	13(22.8)	3(5.3)		
Years Focus on IBD					
≤5 years	110(54.4)	68(33.7)	24(11.9)	13.44	0.098
>5–10 years	79(47)	71(42.3)	18(10.7)		
>10–15 years	69(61.1)	33(29.2)	11(9.7)		
More than 20 years	42(65.6)	19(29.7)	3(4.7)		

#### 3.3.2 Perspectives on 5-ASAUse across hospital levels

**Figure 6:**
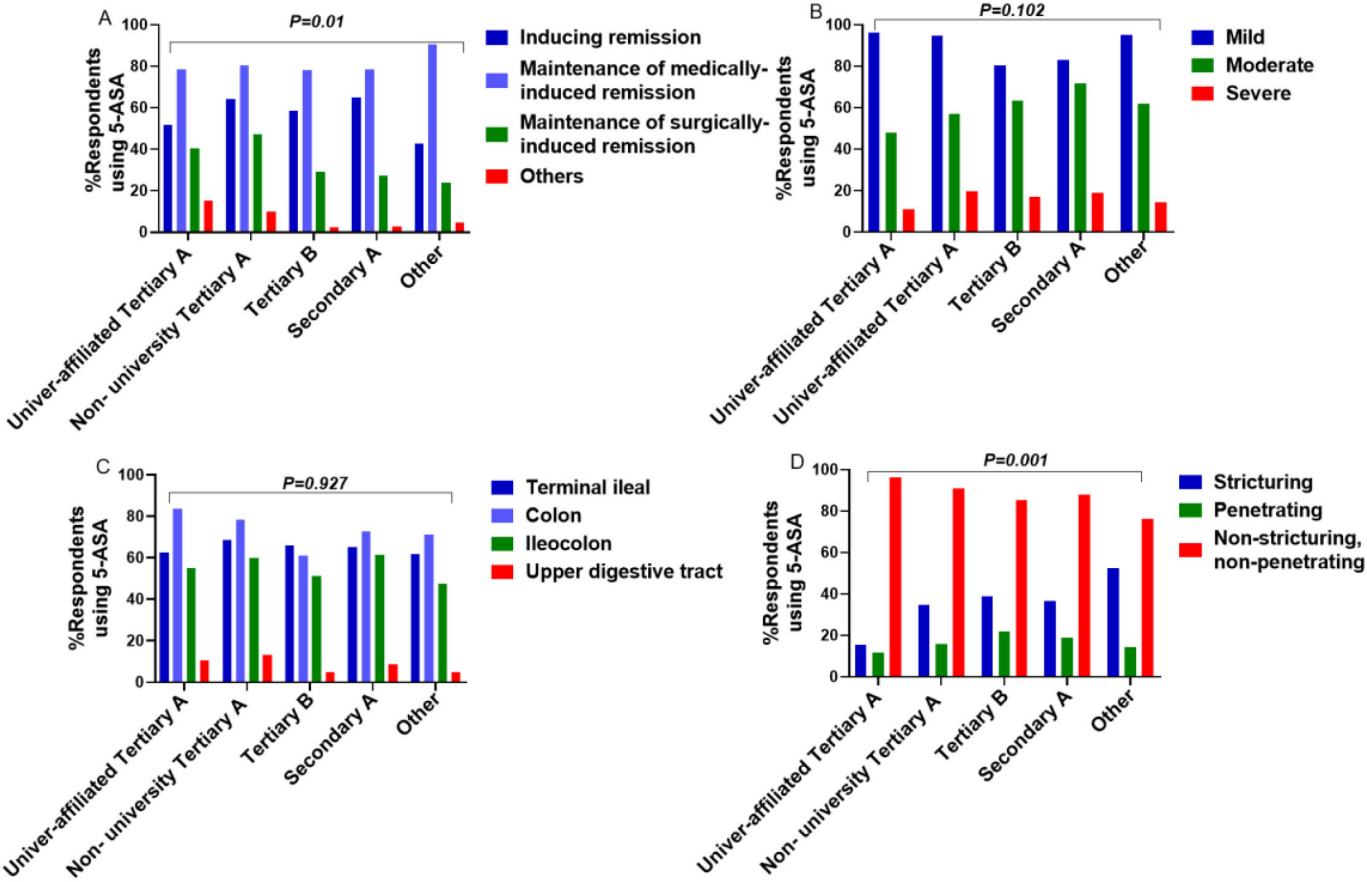
Perspectives on 5-ASA use for CD across hospital levels. **(A)** treatment phase **(B)** disease severity **(C)** disease location **(D)** disease behavior.

Treatment Phase: Significant differences existed in the preferred phase of treatment (induction vs. maintenance) across hospital levels (X²=26.335, P = 0.01) *Post-hoc* analysis (Bonferroni) indicated significant differences between university-affiliated tertiary A hospitals and secondary A (X²=17.109, P = 0.001), suggesting physicians in higher-level hospitals were more likely to restrict use to postoperative maintenance (**Figure 6A**).

Disease Behavior: Significant differences existed in prescribing for different disease behaviors (B1, B2, B3) across hospital levels (X²=25.874, P = 0.001). *Post-hoc* analysis showed significant differences between university-affiliated tertiary A hospitals and non-university tertiary A, secondary A, and other hospitals, indicating a stronger preference for B1 disease in higher-level hospitals (**Figure 6D**).

Disease Severity and Location: No significant differences were found in prescribing based on disease severity or location across hospital levels (Severity: X²=13.305, P = 0.102; Location: X²=5.779, P = 0.927) (**Figures 6B, C**).

#### 3.3.3 Perspectives on 5-ASA use across professional titles

**Figure 7:**
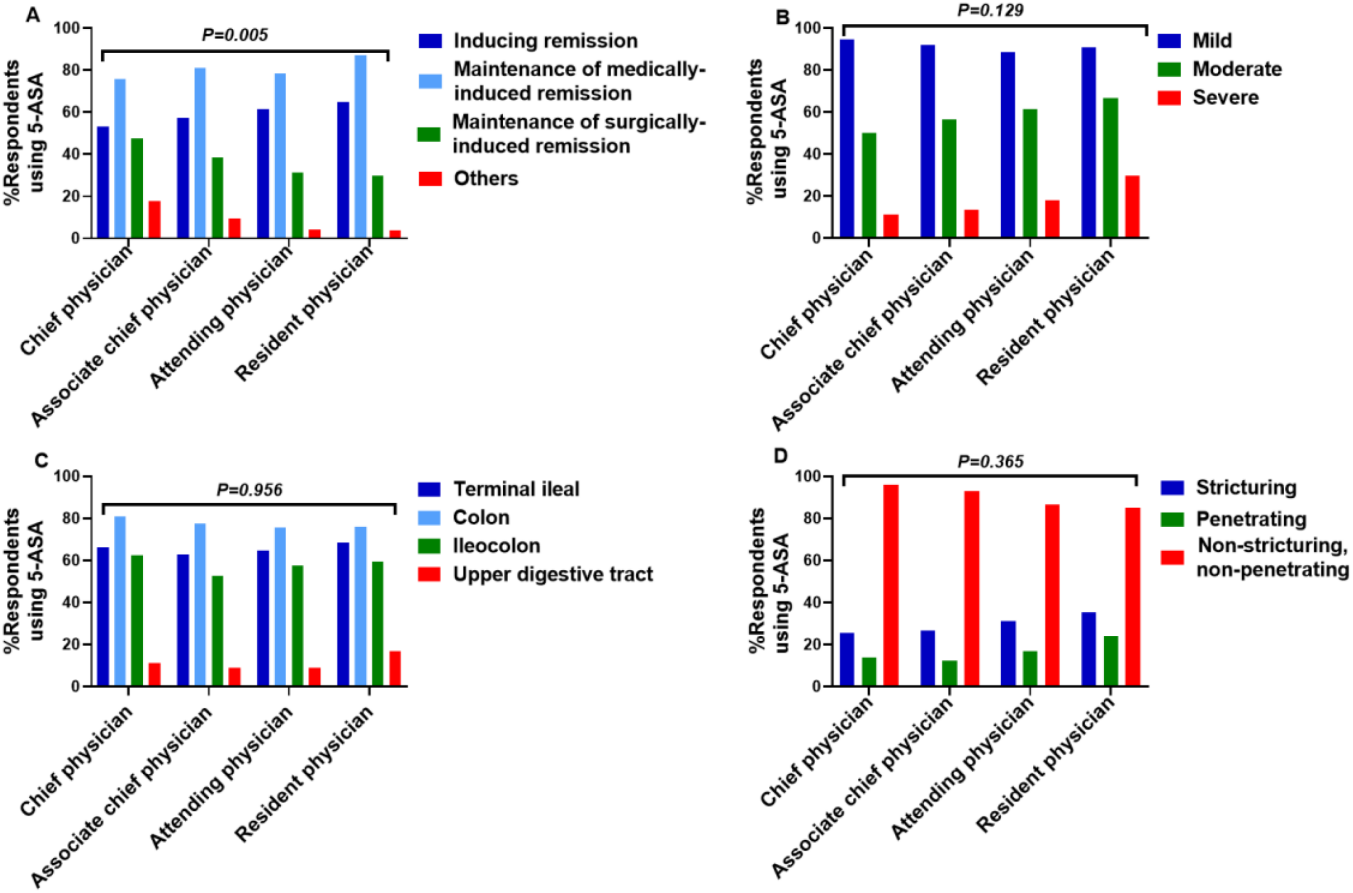
Perspectives on 5-ASA Use for CD across professional titles. **(A)** treatment phase **(B)** disease severity **(C)** disease location **(D)** disease behavior.

Treatment Phase: Significant differences existed in the preferred phase of treatment across professional titles (X²=23.777, P = 0.005). *Post-hoc* analysis indicated a significant difference between Chief Physicians and Attending Physicians (X²=17.412, P = 0.01), suggesting higher-ranking physicians were more likely to restrict use to postoperative maintenance rather than induction (**Figure 7A**).

Disease Severity, Location, Behavior: No significant differences were found in prescribing based on disease severity, location, or behavior across professional titles (Severity: X²=9.892, P = 0.129; Location: X²=3.204, P = 0.956; Behavior: X²=6.543, P = 0.365) (**Figures 7B–D**).

#### 3.3.4 Perspectives on 5-ASA use based on years focused on IBD

No significant differences were found in perspectives on the phase of treatment, disease severity, location, or behavior based on years focused on IBD (Phase: X²=19.927, P = 0.082; Severity: X²=5.194, P = 0.737; Location: X²=5.891, P = 0.921; Behavior: X²=3.765, P = 0.878) (**Supplementary Figure 4**).

#### 3.3.5 Perspectives on induction and maintenance details across hospital levels

##### 3.3.5.1 Induction therapy: dosage and combination

**Figure 8:**
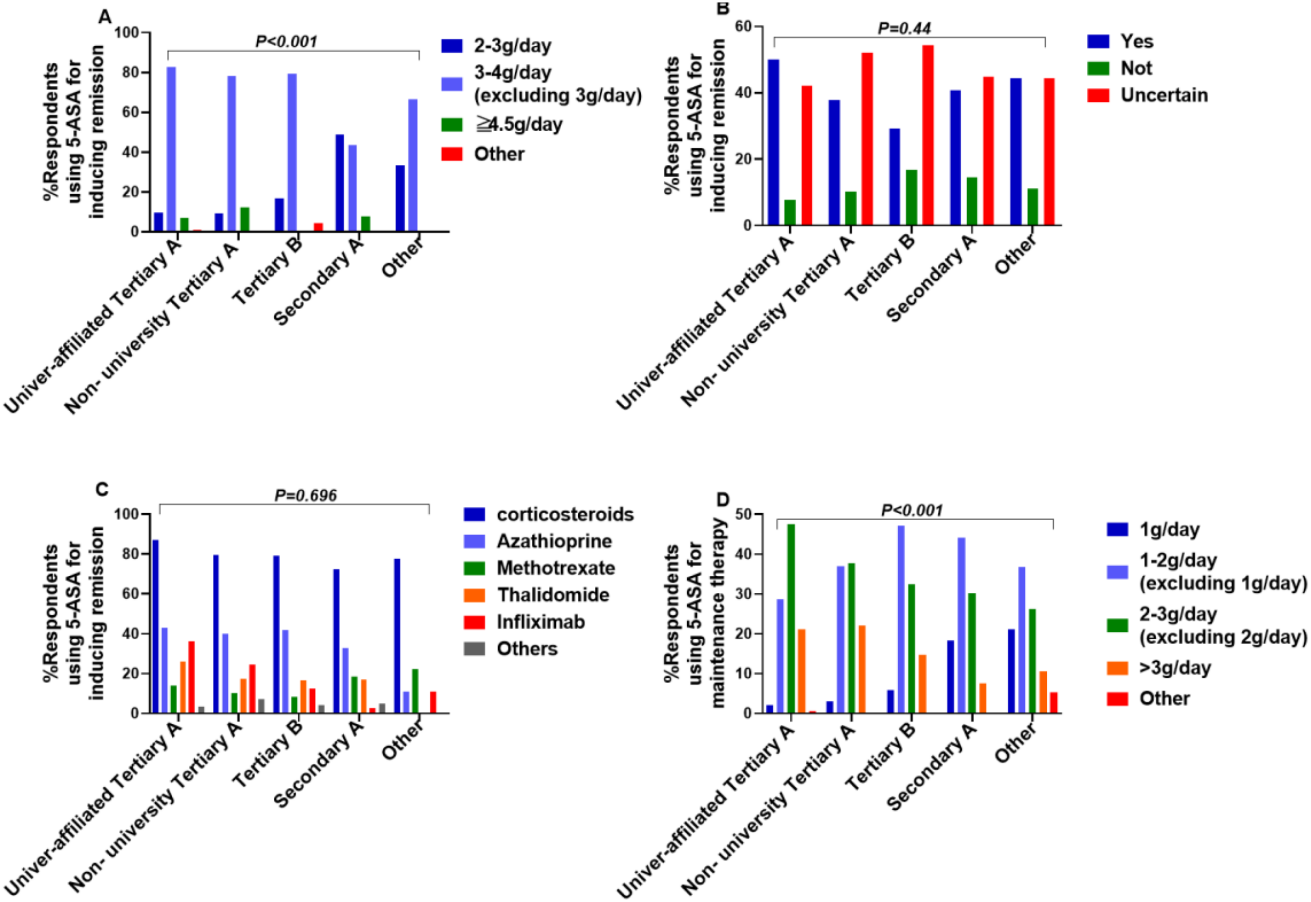
Perspectives on induction and maintenance details across hospital levels **(A)** induction dose **(B)** induction combination decision **(C)** induction combination regimens **(D)** maintenance dose.

Dosage: Significant differences existed in prescribed induction doses across hospital levels (Fisher’s exact test, X²=59.7, P<0.001). Details per level are shown in **Figure 8A**.

Combination Therapy (Decision & Regimen): No significant differences were found in the decision to use combination therapy or the specific regimens chosen across hospital levels (Decision: X²=7.714, P = 0.44; Regimen: X²=15.743, P = 0.696) (**Figures 8B, C**).

##### 3.3.5.2 Maintenance therapy: dosage and combination

Dosage: Significant differences existed in prescribed maintenance doses across hospital levels (Fisher’s exact test, X²=55.396, P<0.001). *Post-hoc* analysis indicated a significant difference between non-university tertiary A hospitals and secondary A (X²=21.581, P<0.001), suggesting a preference for 2–3 g/day(excluding 2 g/day) in higher-level hospitals versus 1–2 g/day(excluding 1 g/day) in lower-level hospitals (**Figure 8D**).

**Figure 9:**
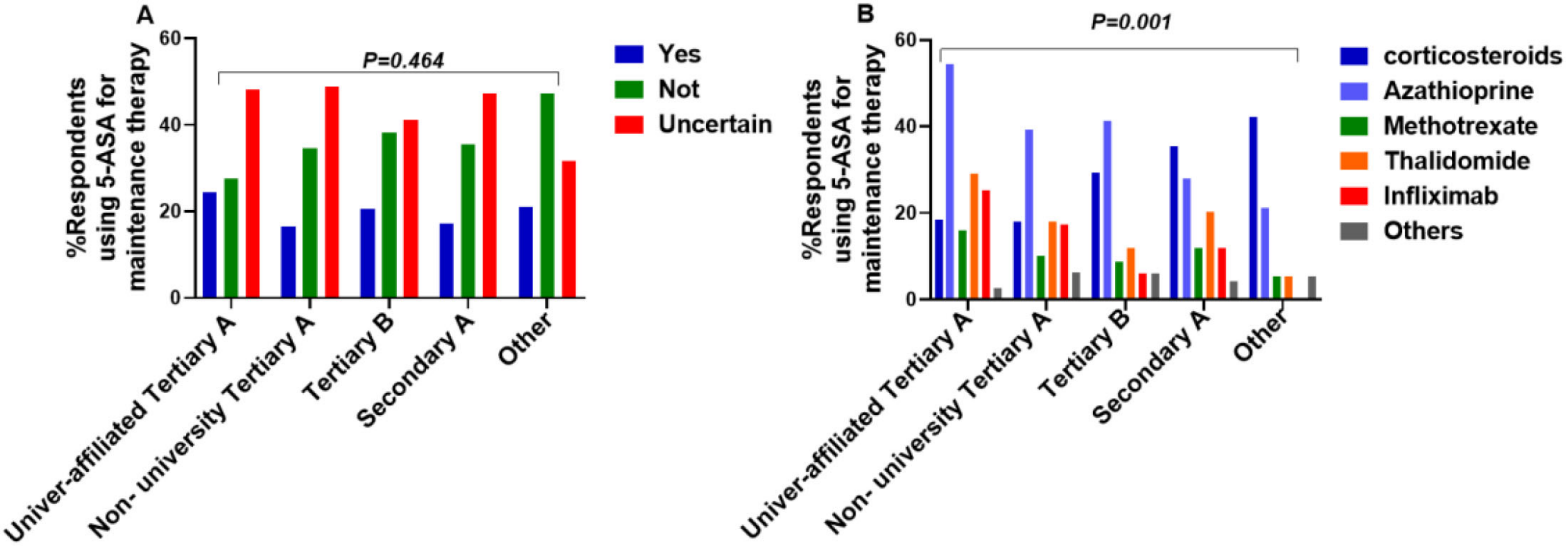
Perspectives on maintenance therapy across hospital levels. **(A)** maintenance combination decision **(B)** maintenance combination regimens.

Combination Therapy (Decision): No significant differences in the decision to use combination therapy for maintenance across hospital levels (X²=7.689, P = 0.464) (**Figure 9A**).

Combination Regimen: Significant differences existed in the choice of combination agents for maintenance across hospital levels (X²=42.357, P = 0.001). *Post-hoc* analysis showed significant differences between university-affiliated tertiary A hospitals and secondary A hospitals/other hospitals, indicating a higher tendency to combine with corticosteroids in lower-level hospitals (**Figure 9B**).

## 4 Discussion

This survey of 615 IBD-focused clinicians in China revealed that 90.4% prescribe 5-ASA for CD patients in clinical practice. Its use is predominant in patients with mild, colonic, non-stricturing non-penetrating disease for induction therapy, and for maintenance therapy following medically-induced remission achieving endoscopic healing. Physicians in higher-level hospitals demonstrated lower rates of 5-ASA use, often restricting it to postoperative maintenance therapy for patients with residual non-stricturing non-penetrating disease. Higher-ranking physicians also showed a stronger tendency to reserve 5-ASA for postoperative maintenance. Regarding maintenance dosing, physicians in higher-level hospitals favored 2–3 g/day (excluding 2 g/day), while those in lower-level hospitals favored 1–2 g/day (excluding 1 g/day) and were more likely to combine it with corticosteroids.

A striking finding of our study is the profound gap between evidence-based guidelines and real-world clinical practice. While the prevailing use of 5-ASA described here is largely unsupported by randomized evidence, it persists pervasively (5–8). The magnitude of this evidence-practice gap in China (90.4%) closely mirrors findings from Western countries, where Ma et al. reported that over 90% of gastroenterologists in Canada, the US, and Europe continue to prescribe 5-ASA for CD patients (13–15). This suggests that the disconnect between evidence and practice is a global phenomenon, not limited to regions with restricted access to advanced therapies.

Clinically, these findings must be situated within the rapidly evolving therapeutic landscape of IBD. Today, biologics and small molecules are increasingly deployed earlier in the disease course to achieve deep remission and mucosal healing. Furthermore, treatment paradigms, such as the use of JAK inhibitors in refractory UC, are continuously shifting expectations regarding efficacy thresholds (16–19). In this context, continuing to rely on low-efficacy drugs like 5-ASA for CD is deeply concerning. This persistent misuse reflects a form of “therapeutic inertia” that offers a false sense of security to both physicians and patients. Crucially, utilizing 5-ASA for CD risks delaying the initiation of truly effective therapies. This delay inevitably increases reliance on systemic corticosteroids, as evidenced by the 90.3% of our respondents who combined 5-ASA with steroids for induction, and allows underlying transmural inflammation to smolder. Ultimately, this undertreatment contributes to irreversible bowel damage, driving complications that necessitate hospitalization and surgical interventions, with all the downstream risks outlined in surgical IBD populations (20–25). Therefore, the persistent use of 5-ASA in CD deserves rigorous clinical critique rather than mere quiet documentation.

The pattern of 5-ASA use observed in this study, predominantly for mild, colonic, non-stricturing non-penetrating disease, reflects a persistent belief among clinicians that certain patient subgroups may benefit, despite the lack of robust evidence from randomized controlled trials. This perception is reinforced by early studies such as the National Cooperative Crohn’s Disease Study (26) and the European Cooperative Crohn’s Disease Study (27), which suggested some benefit for sulfasalazine in colonic disease, and Singleton et al.’s trial showing high-dose mesalamine (4 g/day) superior to placebo for induction (28). However, more recent and methodologically rigorous meta-analyses have consistently failed to confirm these findings (6–8). The 2016 Cochrane review by Lim et al. concluded that high-dose mesalamine (3.2–4 g/day) showed no superiority over placebo for inducing clinical response or remission in active CD (5).

Regarding induction therapy, nearly half of the physicians prescribed 3–4 g/day(excluding 3 g/day). The high rate of combination therapy (89.2%), with 90.3% combining with corticosteroids, indirectly suggests that clinicians lack confidence in 5-ASA monotherapy. While corticosteroids are effective for inducing symptomatic remission in CD (9, 10), combining them with an ineffective agent exposes patients to the side effects of both drugs without additional therapeutic benefit. This practice pattern may reflect historical treatment algorithms rather than current evidence-based recommendations.

For maintenance therapy, we found 79.5% of physicians using 5-ASA for post-remission maintenance and 38.1% for postoperative maintenance. Cochrane reviews indicate no benefit for 5-ASA in maintaining medically-induced remission, while showing marginal benefit over placebo for postoperative maintenance, albeit with a high Number Needed to Treat (NNT = 13) (7, 8). Even in the postoperative setting, the modest benefit must be weighed against the availability of more effective agents. Combining 5-ASA with azathioprine (AZA) for CD maintenance is common in practice. However, clinicians must be aware of the potential drug-drug interaction when combining 5-ASA with azathioprine: 5-ASA inhibits thiopurine methyltransferase (TPMT), potentially leading to significantly increased levels of azathioprine’s active metabolite, 6-thioguanine (6-TGN) (29), thereby increasing the risk of myelosuppression (30). This interaction is often overlooked in clinical practice.

Regarding reasons for choosing 5-ASA, 75.5% cited “good safety and low toxicity.” While 5-ASA agents do have a milder side effect profile compared to corticosteroids and thiopurine (31), the risk-benefit calculus must consider therapeutic efficacy. Prescribing a safe but ineffective drug exposes patients to the risks of untreated disease without conferring benefit. The perception of “relatively lower cost” (cited by 63.1%) also warrants scrutiny. A health cost study in Victoria showed that 5-ASA accounted for 15% of total direct CD costs (32), and when considering the costs of disease progression, hospitalization, and surgery resulting from inadequate treatment, the economic argument for 5-ASA becomes less compelling.

The finding that physicians in higher-level hospitals and those with higher professional titles demonstrated lower rates of 5-ASA use and were more likely to restrict it to postoperative maintenance is encouraging. This likely reflects those physicians can better access to continuing medical education, greater exposure to updated guidelines, and more opportunities to participate in multidisciplinary discussions. Conversely, the higher rates of 5-ASA use in lower-level hospitals, particularly in combination with corticosteroids, highlight an urgent need for targeted educational interventions at these institutions.

Perspectives on 5-ASA side effects largely align with existing data (33). Gastrointestinal reactions were deemed most common (62.2%), followed by abnormal liver function (52.5%). Physicians’ assessment of adherence to 6 months of 5-ASA therapy was pessimistic, with only 20.7% estimating ≥75% adherence, consistent with international studies reporting poor adherence (40-60%) in CD patients prescribed 5-ASA (34–36).

This study has several limitations. First, response bias is likely substantial. Physicians who actively prescribe and believe in the utility of 5-ASA may have been more inclined to complete the survey, potentially overestimating the national prevalence of its use. Second, the survey relied on self-report rather than objective prescribing data, which may be subject to social desirability bias. Third, as noted in the methodology, the lack of a standardized definition for “mild” CD means that interpretation varied across different regions, hospital levels, and physician training backgrounds. What constitutes “mild” disease in a secondary hospital may differ significantly from that in a university-affiliated tertiary center. Fourth, institutional clustering may have occurred, though we attempted to minimize this by limiting responses per institution. Fifth, the cross-sectional design captures practice patterns at a single time point and cannot assess changes over time. Sixth, the generalizability of findings to non-responders and to regions not well-represented in the WeChat groups is uncertain.

In summary, this study provides the first confirmation of the widespread use of 5-ASA for CD among Chinese clinicians, highlighting a significant gap between real-world practice and guideline recommendations. The core message must be stated clearly: 5-ASA has a well-established role in UC but lacks evidence of meaningful efficacy in CD. The persistent use of 5-ASA in CD, documented globally including in this Chinese cohort, represents therapeutic inertia that may delay effective treatment, increase steroid exposure, and ultimately contribute to disease complications. Addressing this evidence-practice gap requires comprehensive, systematic continuing education for clinicians, particularly those in lower-level hospitals, and effective channels to disseminate accurate information about chronic disease management to both physicians and patients. Future research should evaluate whether targeted educational interventions can successfully align prescribing practices with current evidence and improve patient outcomes.

## Data Availability

The raw data supporting the conclusions of this article will be made available by the authors, without undue reservation.
