# Supplementary material for: Current status of 5-aminosalicylic acid in Crohn’s disease treatment: a nationwide survey and analysis of perspectives and practice patterns among inflammatory bowel disease physicians
Source: Front Gastroenterol (Lausanne). 2026 Apr 27;5:1783772. doi: 10.3389/fgstr.2026.1783772 (PMC13158092; doi:10.3389/fgstr.2026.1783772)
Supplement: Supplementary file 1 [file Table1.docx]

**Current Status of 5-Aminosalicylic Acid (5-ASA) in Crohn's Disease Treatment: A Nationwide Survey and Analysis of Perspectives and Practice Patterns Among Inflammatory Bowel Disease (IBD) Physicians**

This questionnaire is anonymous. Your answers are not "right, wrong, good, bad", only for research and analysis. Your truthful answers are the basis of our research success. Thank you sincerely for your support and cooperation!

I confirm that I have read the above information and voluntarily agree to participate in this survey.

**1 Basic information of doctors**

- 1. The level of your hospital

A Grade IIIA Hospital directly under the medical universities

B Grade IIIA Hospital directly under non-medical universities

C Grade IIIB Hospital

D Grade II A Hospital

E Others

- 1. Your profession

A Gastroenterology Department

B Anorectal surgery

C Pediatrics Department

D Others

- 1. Your professional title

A Chief physician

B Associate chief physician

C Attending physician

D Resident

- 1. Years of focus on IBD work

A ≤5 years

B >5-10 years

C >10-15 years

D >15-20 years

E More than 20 years

- 1. Where are you from?

A North China

B Northeast China

C East China

D Central China

E South China

F Southwest China

G Northwest China

1. **Treatment of Crohn’s disease**
   1. Do you think 5-aminosalicylic acid(5-ASA) is effective for Crohn's disease(CD)?

A Effectiveness

B Ineffectiveness

C Possible effectiveness

- 1. What circumstances do you usually use 5-ASA for? (multiple choices) (depending on options 1 and 2 of question 2.1)

A Inducing remission

B Maintenance of medically-induced remission

C Maintenance of surgically-induced remission

D Other conditions

- 1. What severity of Crohn's disease do you use 5-ASA for? (multiple choices) (depending on options 1 and 2 of question 2.1)

A Mild

B Moderate

C Severe

- 1. Where do you usually use 5-ASA in? (depending on options 1 and 2 of question 2.1)

A Terminal ileal

B Colon

C Ileocolon

D Upper digestive tract

- 1. What disease behavior of CD do you use 5-ASA for? (depending on options 1 and 2 of question 2.1) (multiple choices)

A Stenotic

B Penetrating

C Non-stenotic, non-penetrating

- 1. Which 5-ASA preparation do you usually use (depending on options 1 and 2 of question 2.1)

A Sulfasalazine

B Balsalazide

C Olsalazine

D Mesalamine sustained-release granules

E Mesalamine sustained-release tablets

F Mesalamine enteric-coated tablets

1. **Inducing remission**
   1. What dose of 5-ASA do you use for induction remission therapy? (depending on option 1 of question 2.2)

A 2 to 3 g/day

B 3 to 4 g/day (excluding 3 g)

C ≧ 4.5 g/day

D Other

- 1. For inducing remission, do you use drugs in combination? (depending on option 1 of question 2.2)

A Yes

B Not

C Uncertain

- 1. If you have experience in combination, what medications are typically used in combination for induction of remission? (multiple choice) (depending on options 1 and 3 on question 3.2)

A Corticosteroids

B Azathioprine

C Methotrexate

D Thalidomide

E Infliximab

F Other

1. **Maintenance therapy**
   1. What is your dose of 5-ASA for maintenance therapy? (depending on option 2 and 3 of question 2.2)

A 1g/day

B 1 to 2g/day (excluding 1g)

C 2 to 3g/day (excluding 2g)

D >3g/day

E Other

- 1. For maintenance therapy, do you use drugs in combination? (depending on option 2 and 3 of question 2.2)

A Yes

B Not

C Uncertain

- 1. If you have experience in combination, what medications are typically used in combination for maintenance therapy? (multiple choice) (depending on options 1 and 3 on question 4.2)

A Corticosteroids

B Azathioprine

C Methotrexate

D Thalidomide

E Infliximab

F Other

**5 Other**

5.1 What are the common adverse reactions in CD patients treated with 5-ASA (multiple choices) (depending on options 1 and 2 of question 2.1)

A Nervous system such as dizziness and headache

B Skin rash

C Gastrointestinal reactions such as abdominal pain and diarrhea

D Allergy

E Blood system problems

F Abnormal liver function

G Renal damage

H Others

5.2 What do you think of your patient's compliance for half a year during 5-ASA treatment? (depending on options 1 and 2 of question 2.1)

A ≤25% of the time

B >25%-50% of the time

C >50%-75% of the time

D ≧75% of the time

E Uncertain

5.3 Why do you support the use of 5-ASA in CD patients? (depending on options 1 and 2 of question 2.1)

A There was evidence of efficacy in mild disease and in patient subgroups not subjected to randomized controlled trials.

B 5-ASA was relatively low cost (especially compared with biological agents)

C 5-ASA had good safety and low toxicity

D 5-ASA might prevent colon cancer associated with inflammatory bowel disease

E Myself clinical experience.

5.4 Why do you not support the use of 5-ASA in CD patients? (depending on the option 3 of question 2.1)

A There was high-quality evidence that 5-ASA had a slight therapeutic advantage in inducing and maintaining remission (compared with placebo)

B 5-ASA have rare and severe adverse reactions.

C Other therapies had a higher chance of inducing remission and improving quality of life.

D Others

Figure S1 Physicians' perspectives on the efficacy of 5-ASA for CD

Figure S2 Types of CD for which Physicians Prescribe 5-ASA (This was a multiple-choice question; therefore, the sum of percentages exceeds 100%.)

Figure S3 5-ASA Formulations Prescribed by Physicians (This was a multiple-choice question; therefore, the sum of percentages exceeds 100%.)

**
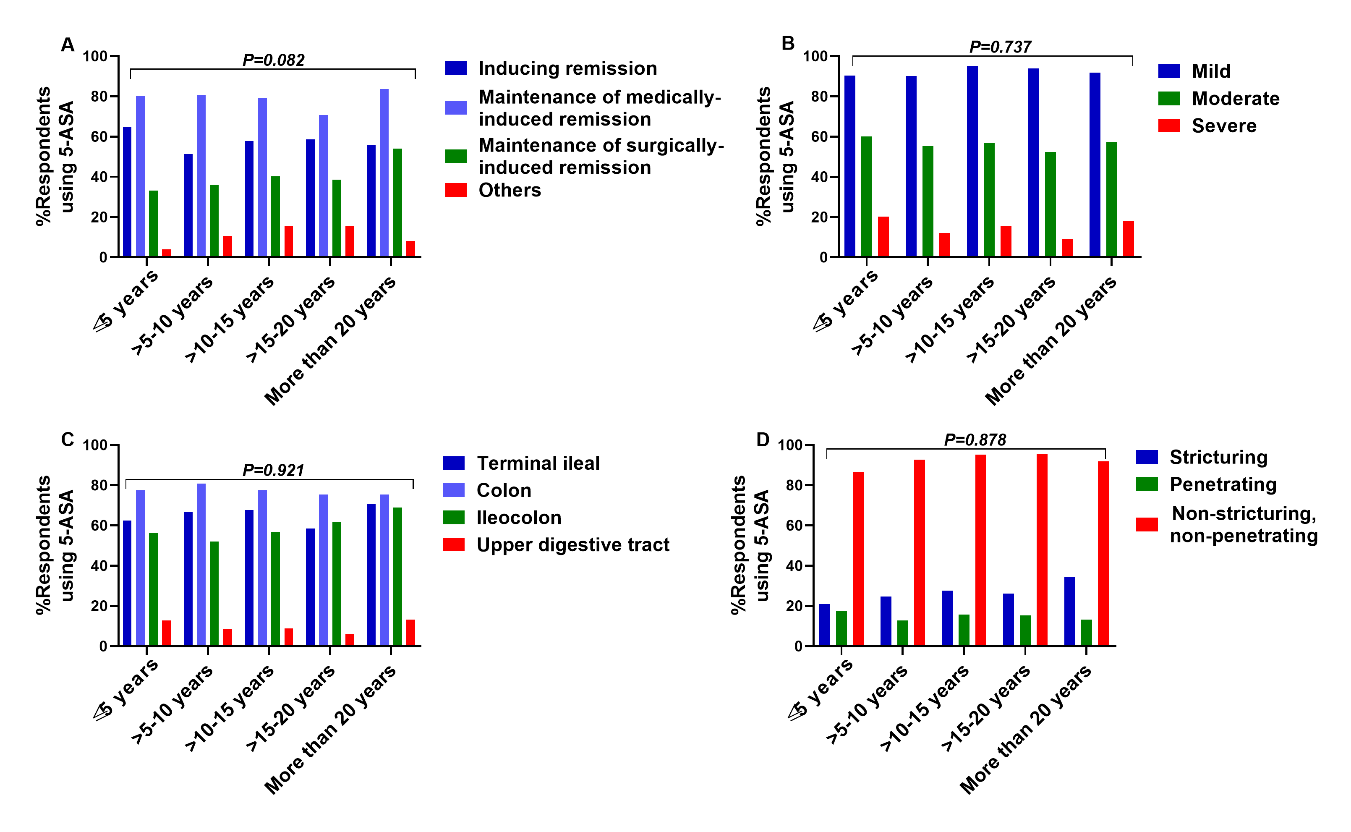
**

Figure S4 Perspectives on 5-ASA Use for CD Based on Years Focused on IBD

(A) Treatment Phase (B) Disease Severity (C) Disease Location (D) Disease Behavior
